# Diagnosis of placental malaria in poorly fixed and processed placental tissue

**DOI:** 10.1186/s12936-016-1314-6

**Published:** 2016-05-10

**Authors:** Yunhao Liu, Jennifer B. Griffin, Atis Muehlenbachs, Stephen J. Rogerson, Anya J. Bailis, Rajni Sharma, David J. Sullivan, Antoinette K. Tshefu, Sarah H. Landis, Jean-Marie M. Kabongo, Steve M. Taylor, Steven R. Meshnick

**Affiliations:** Department of Epidemiology, UNC Gillings School of Global Public Health, Chapel Hill, NC USA; Department of Statistics and Operations Research, University of North Carolina, Chapel Hill, NC USA; RTI International, Research Triangle Park, NC USA; Department of Pathology, University of Washington, Seattle, WA USA; Department of Medicine at Peter Doherty Institute, University of Melbourne, Melbourne, Australia; Division of Maternal–Fetal Medicine, Department of Gynecology & Obstetrics, Johns Hopkins University School of Medicine, Baltimore, MD USA; Immunopathology Laboratory, Johns Hopkins University School of Medicine, Baltimore, MD USA; W. Harry Feinstone Department of Molecular Microbiology and Immunology, Bloomberg School of Public Health, Johns Hopkins University, Baltimore, MD USA; Kinshasa School of Public Health, Kinshasa, Democratic Republic of the Congo; Glaxo-Smith-Kline, Worldwide Epidemiology, Uxbridge, Middlesex, UK; Department of Medical Biology, Service of Pathology, University of Kinshasa Medical School and University Hospital, Kinshasa, Democratic Republic of the Congo; Division of Infectious Diseases & International Health and Duke Global Health Institute, Duke University Medical Center, Durham, NC USA

**Keywords:** Malaria, Histopathology, PCR, IHC, Latent class analysis, Molecular epidemiology

## Abstract

**Background:**

Placental histopathology has been considered the gold standard for diagnosis of malaria during pregnancy. However, in under-resourced areas placental tissue is often improperly fixed and processed; the resulting formalin pigment is difficult to distinguish from malaria pigment. This study examines two alternative diagnostic methods: polymerase chain reaction (PCR) and a novel immunohistochemistry (IHC)-based method using an antibody against histidine-rich protein 2 (HRP2).

**Methods:**

Placental histopathology from 151 pregnant women in Kinshasa was assessed by two blinded microscopists and compared with peripheral blood PCR and IHC for HRP2. The Cohen’s kappa coefficients were calculated to assess the test agreement. The sensitivity and specificity of individual tests were calculated using PCR or IHC as the reference standard as well as latent class analysis (LCA).

**Results:**

PCR and IHC correlated fairly well. The correlation between the two blinded microscopists was poor, as there was widespread formalin pigment. Using LCA, all of the tests had high specificities. The most sensitive test was IHC (67.7 %), with PCR as second-best (56.1 %).

**Conclusions:**

PCR and/or IHC are suitable diagnostics when the presence of formalin pigment substantially compromises placental histopathology.

## Background

Globally, there are 85.3 million pregnancies occurring annually in *Plasmodium falciparum*-endemic areas [1, 2]. Malaria infection in pregnancy has significant, direct, negative health effects on the mother and the neonate, such as severe maternal anaemia and related maternal mortality, low birth weight from both preterm delivery and intra-uterine growth restriction, and postnatal mortality [3]. A key risk factor for these complications is placental malaria, defined as the sequestration of *P. falciparum*-infected erythrocytes in the placenta. The ability to accurately identify placental malaria before and at delivery is critical for both research and programmatic purposes.

It is often difficult to diagnose malaria in pregnant women because parasitized erythrocytes sequestered in the placenta are often absent from the peripheral circulation. Microscopy of peripheral and placental blood, polymerase chain reaction (PCR), and rapid diagnostic tests have been used but vary in sensitivity and specificity. Traditionally, histopathology has been the gold standard for the diagnosis of placental malaria (reviewed in [4, 5]). The quality of histopathology is very dependent on obtaining high-quality specimens, since formalin pigment has similar optical properties to malaria pigment and can lead to misclassification [6]. Unfortunately, residual formalin pigment is common in under-resourced areas due to the use of inexpensive, low-quality fixative.

Here PCR, immunohistochemistry (IHC) and histopathology are compared as diagnostics for *falciparum* malaria using dried blood spots and formalin-fixed placental tissue from a longitudinal cohort study conducted in Kinshasa, Democratic Republic of Congo (DRC) in 2005–2006 [7, 8]. Because the placental tissue had abundant formalin pigment, two separate microscopists examined them. The aim of this analysis is to examine the concordances of the different tests using both the classical contingency table [9] and the latent class analysis (LCA) approaches [10]. LCA can be used to estimate the sensitivity and specificity for independent diagnostic tests, when all tests are regarded as imperfect.

## Methods

### Study population

A prospective study conducted among women seeking antenatal care at a maternity hospital in Kinshasa, DRC examined the effect of malaria in pregnancy on intra-uterine growth restriction. From May 2005 to May 2006, 182 adult women with healthy, singleton pregnancies less than 23 weeks gestational age were enrolled and followed longitudinally until delivery [7]. Pregnant women received intermittent preventive therapy with two doses of sulfadoxine-pyrimethamine (SP) and an insecticide-treated bednet in accordance with DRC national guidelines. Women who presented for unscheduled visits and were parasitaemic were treated with SP. Enrolled women provided written informed consent at enrollment.

This study was approved by the Institutional Review Boards of the University of North Carolina at Chapel Hill (UNC) and the Kinshasa School of Public Health.

### Clinical data and specimen collection

At monthly follow-up visits and at delivery, peripheral blood was collected and applied to Schleicher and Schuell 903 specimen paper. Due to the large number of missing peripheral blood samples from delivery, the final antenatal dried blood spot obtained (usually in the late third trimester) was used. After delivery, a placental biopsy was collected from an incision at a healthy pericentric area of the placenta. Following the procedures described by Rogerson [11], two placental biopsy samples (approximately 1 cu cm) were taken and placed into 10 % neutral buffered formalin. Samples were stored for no longer than a month until sent to the University of Kinshasa *Service d’Anatomie Pathologique*, where they were embedded in paraffin using the standard techniques and the first histopathological analysis performed.

### PCR of antenatal peripheral blood

Blood samples were collected from study participants 0 to 33 days before delivery with one exception (one woman gave blood 77 days before delivery). Real-time quantitative PCR (qPCR) was performed at UNC on DNA extracted from filter paper-dried blood spots using a high-throughput pooling strategy as previously described by Taylor et al. [8]. Briefly, genomic DNA was tested in qPCR assays targeting the *Plasmodium* spp 18S rRNA sub-unit using TaqMan probes; in an initial reaction, primers and probes detected any species of *Plasmodium*; in two subsequent duplex reactions, primers and probes detected *P. falciparum, P. ovale, P. malariae*, and a human control gene. The assay detected gDNA from stock strains of *P. falciparum* and *P. vivax* at a concentration of 0.001 ng/μl (39 parasites per μl, based on an estimated genome size of 23 Mb), with an average CT value of 32.63 (SD, 0.008).

### Histopathology for placental malaria

At UNC, paraffin sections of approximately 5 µm were stained with Giemsa according to standard histologic protocols [12]. The stained sections were examined by two blinded microscopists under light microscopy to assess parasitaemia and malaria pigment. Sections were examined under oil immersion or using a dry 60× objective for the presence of parasitized erythrocytes and malaria pigment in fibrin using the classification system previously described by Bulmer et al. [13].

### IHC for histidine-rich protein 2

IHC using monoclonal antibody 3A4 [14, 15] to *P. falciparum* histidine-rich protein 2 (HRP2) was carried out on 5 mm thick, formalin-fixed, paraffin-embedded sections using automated IHC stainer (Bond Leica, Leica Microsystems, Bannockburn, IL, USA) at the Johns Hopkins Immunopathology Research Laboratory as previously described [16]. Slides were deparaffinized and hydrated, and heat-induced antigen retrieval step was performed using citrate buffer (pH 6.0). Before antibody incubation a 3 % hydrogen peroxide solution for 5 min blocked endogenous peroxidase. Incubation with the primary antibody using optimal conditions (dilution 1:400) was followed by incubation with secondary antibody and red detection. The secondary antibody and biotin-free polymer red detection was applied per manufacturer instructions (code #DS9390- Bond Polymer Refine Red Detection, Leica Microsystems, Bannockburn, IL, USA). A counterstaining with haematoxylin was applied, and slides were dehydrated and cover slipped.

### Data analysis

Data collected in the DRC were entered into databases created in EpiInfo (Centers for Disease Control and Prevention, Atlanta, GA, USA). Laboratory data were entered into databases created in Microsoft Excel 2000 (Microsoft, Redmond, WA, USA). All databases were imported into SAS for data cleaning and management. Analyses were performed with SAS version 9.4 (SAS Institute, Cary, NC, USA). The Cohen’s kappa coefficients for pairs of diagnostic tests were computed using the PROC FREQ procedure of SAS. The sensitivity and specificity of individual tests were calculated using both a dichotomy format contingency table [9] and the Proc LCA package for SAS [17] (The Methodology Center, Pennsylvania State University, PA, USA) by following the previously described procedures [17]. The results from Proc LCA were confirmed using the poLCA package for R (R Foundation for Statistical Computing, Vienna, Austria).

## Results

### Study sample

Of 182 women in the study, 151 placental biopsy specimens were available for the current analysis. Table 1 describes characteristics of the study subjects; details of the full study have been published previously [7]. The ages of the pregnant women ranged from 18 to 43 years, with a median of 27 years. About 23 % of women were primigravidae and 15 % secundigravidae. Four participants (2.6 %) were HIV-positive. The prevalence of malaria determined by the different microscopists and methods is shown in Table 2. Approximately 29 % of the participants were qPCR-positive for peripheral malaria parasitaemia at least once during pregnancy. The prevalence of malaria parasitaemia based on qPCR of peripheral blood decreased over the course of pregnancy from 21 % during the early second trimester to 9 % during the late third trimester. Nearly all infections were sub-clinical, with only one case having concurrent fever.

**Table 1:** Characteristics of 151 study participants in Kinshasa, DR Congo, 2005–2006

	Number (%)
Maternal age (years)	
Median	27 (−)
18–24	46 (30.5)
25–29	53 (35.1)
≥30	52 (34.4)
Gravidity	
Primigravidae	35 (23.2)
Secundigravidae	22 (14.6)
Multigravidae	94 (62.3)
HIV-positive	4 (2.6)
Ever parasitaemic^a^	44 (29.1)

^a^ By qPCR of *P. falciparum* from dried blood spots

**Table 2:** Prevalence of malaria as determined by different microscopists and methods

Method	# Positive (n = 151)	% Prevalence
Histopathology by P1	5	3.31
Histopathology by P2	10	6.62
IHC	14	9.27
PCR	8	5.30
LCA	–	6.71

**Fig. 1:**
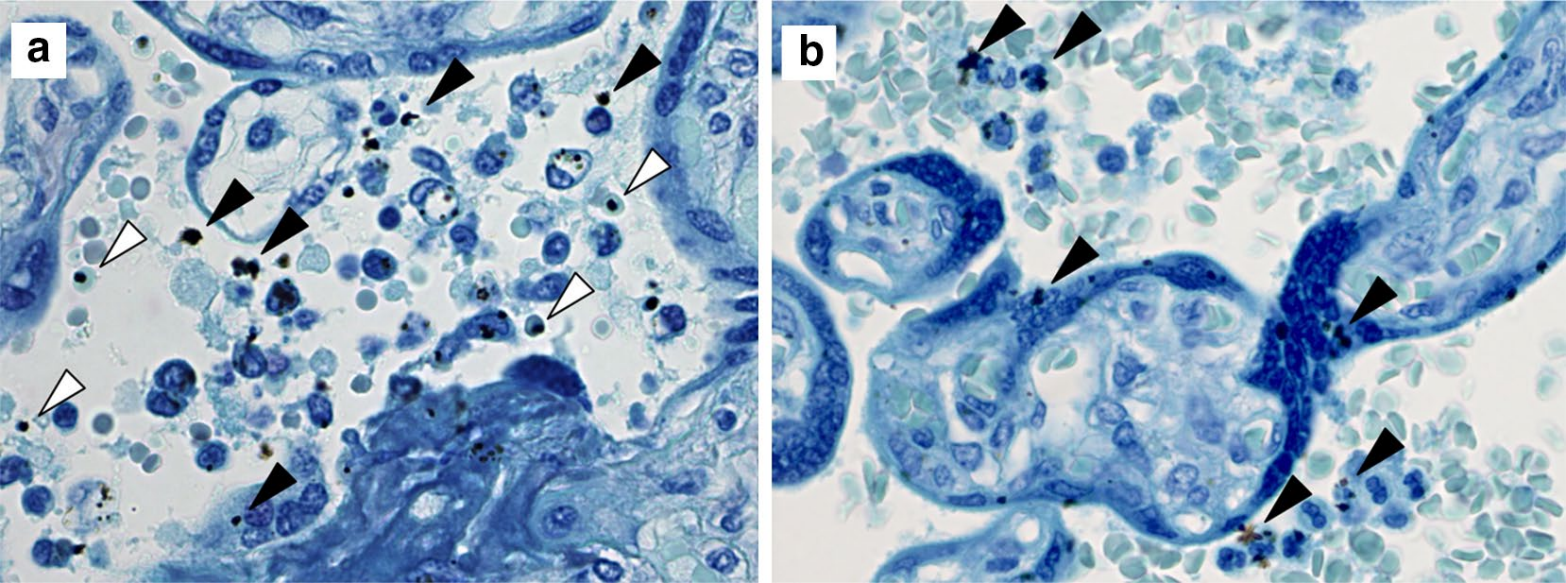
Parasites and formalin pigment in Giemsa-stained placental sections from this study. **a** Placenta with parasitized erythrocytes (*empty arrowheads*) and formalin pigment (*filled arrowheads*). **b** Placenta with formalin pigment (*filled arrowheads*) and no parasitized erythrocytes identified. Original magnifications: ×600

**Fig. 2:**
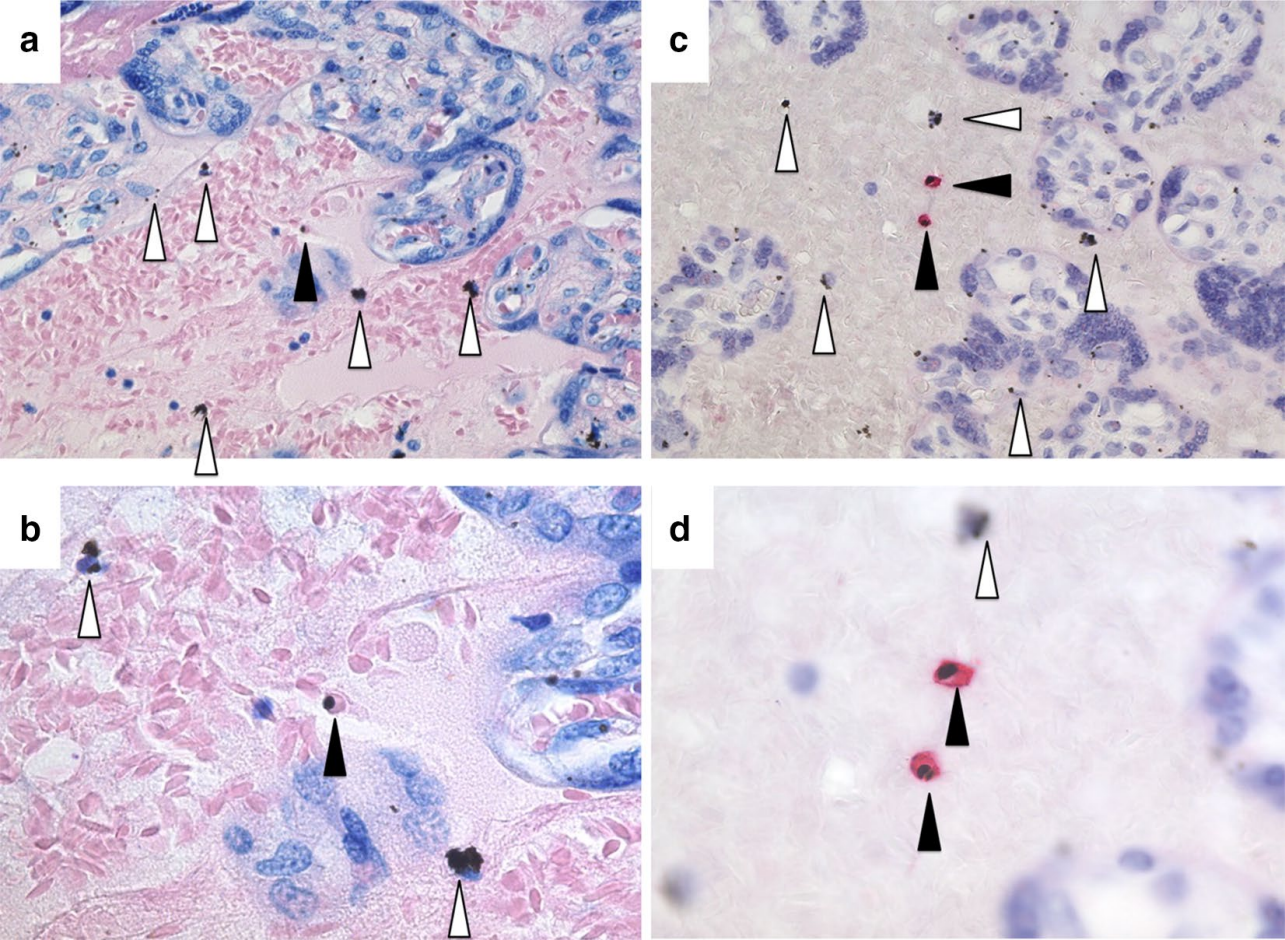
Comparison of Giemsa and IHC under two different magnifications. A low parasitaemic placental sample was stained by Giemsa (**a**, **b**) and by IHC (**c**, **d**) and photographed under two magnifications (erythrocytes are 6 μ in diameter). Under higher magnifications of Giemsa-stained samples, the intra-erythrocytic malaria pigment (*filled arrowheads*) can be distinguished from formalin pigment (*empty arrowheads*) while by IHC the parasites are visible under low magnification. Images were made from placental biopsies used as positive control for this IHC obtained in a previously published study in Uganda [16]

All placental biopsy specimens had a moderate to high level of formalin pigment deposition (Fig. 1). In order to be able to better visualize parasites, IHC was used. This method stains parasite-associated HRP2 (Fig. 2).

### Correlations among tests

The Cohen’s kappa is a statistic that measures the agreement between diagnostic tests [18, 19]. As multiple tests were conducted in this study, the Cohen’s kappa coefficients were calculated to evaluate the correlations between each pair of conducted tests. As shown in Table 3, the overall agreement among the tests ranged from poor to moderate. There was poor correlation between the two histopathology readers. It is noteworthy that during the study, both pathologists suggested that the quality of the paraffin sections was low and reading results off them was not reliable.

**Table 3:** Cohen’s kappa coefficients between the diagnostic tests

Tests chosen to compute the κ coefficient		*κ* value	*p* value for *H*_*0*_: *κ* = 0
Test 1	Test 2		
Histopathology by P1	Histopathology by P2	−0.0462	0.5448
Histopathology by P1	IHC	0.0594	0.4002
Histopathology by P1	PCR	−0.0425	0.5907
Histopathology by P2	IHC	0.2775	0.0005*
Histopathology by P2	PCR	0.2916	0.0003*
IHC	PCR	0.3176	*<*0.0001*

P1 and P2 denote two blinded microscopists

* *p* value < 0.01

The histopathology result from one reader (P1) also correlated poorly with PCR or IHC. Readings by P2, on the other hand, correlated fairly well with IHC and PCR. Agreement between IHC and PCR was fairly good as well.

### Sensitivity and specificity of diagnostic methods

The sensitivity and specificity of diagnostic tests using different reference standards were determined. First, PCR (at time closest to delivery) was used as the gold standard (Table 4). With PCR as the reference standard, all of the other tests had high specificities, but low sensitivities. The most sensitive other test was IHC, but it missed 50 % of the malaria infections diagnosed by PCR. Second, IHC was used as the reference standard (Table 5). Surprisingly, with IHC as the referent, both histopathology (P2) and PCR showed poor sensitivity, missing >70 % of infections. Finally, LCA was used to develop an “alloyed” gold standard (Table 6). LCA takes into account the results of all of the tests in determining the reference standard. All four tests had relatively high specificities, but on sensitivity, IHC slightly outperformed PCR and histopathology by reader P2.

**Table 4:** Sensitivity and specificity of diagnostic tests for placental malaria using qPCR as the criterion standard

	% Sensitivity	% Specificity
Histopathology by P1	0	96.5
Histopathology by P2	37.5	95.1
IHC	50.0	93.0

**Table 5:** Sensitivity and specificity of diagnostic tests for placental malaria using IHC as the criterion standard

	% Sensitivity	% Specificity
Histopathology by P1	7.1	97.1
Histopathology by P2	28.6	95.6
PCR	28.6	97.1

**Table 6:** Latent class analysis of assays for the detection of placental malaria

Test	% Sensitivity (95% CI)	% Specificity (95% CI)
Histopathology by P1	0 (−)	96.5 (93.3–99.6)
Histopathology by P2	54.4 (3.03–100)	96.8 (93.0–100)
IHC	67.7 (10.5–100)	94.9 (90.2–99.7)
PCR	56.1 (2.66–100)	98.4 (95.0–100)

*CI* confidence interval

## Discussion

Under ideal circumstances, placental histopathology is considered the gold standard for diagnosis of placental malaria [4, 5]. However, in this study, both histopathologists noted an abundance of formalin pigment, which makes accurate diagnosis difficult. Because of this, the correlation between the two blinded pathologists was poor. Here, alternative diagnostic approaches were evaluated, including IHC and PCR. Because of the absence of a gold standard, sensitivity and specificity were evaluated in several different ways.

The extent of formalin pigment in this cohort was a surprise considering the use of buffered formalin and careful storage conditions. A possibility is that low-quality formalin was used in the first step of the hospital processor during the paraffin embedding process (formalin is often used to continue fixation of recently grossed specimens). Researchers should be aware of this potential pitfall. Other ways to avoid formalin pigment are to use fresh neutral buffered formalin, small biopsy size with >10:1 ratio of formalin to sample in container, prevent desiccation, and prompt processing or prompt transfer to 70 % ethanol after 24–48 h of fixation.

Correlation between methods was first measured using Cohen’s kappa [18, 19]. Using this method, the findings of the two microscopists did not correlate at all. There was some correlation between one of the pathologists (P2), and both IHC and PCR. Similarly, there was some correlation between IHC and PCR. This is consistent with the finding that none of the tests had sensitivities >50 % compared to IHC or PCR.

Some of the discordances are expected, since different tests have different operator characteristics. For example, the 50 % sensitivity of IHC compared to PCR could have been due to the fact that PCR detects parasite densities that are lower than the threshold for microscopic detection. Alternately, the discordance could have also been the result of timing: the PCRs were done at the last antenatal visit and could have cleared by the time of delivery. On the other hand, PCR only had a 29 % sensitivity compared to IHC. This could be because parasitized erythrocytes sequester in the placenta [20]; thus detectable placental infections are often associated with undetectable peripheral infections.

The current study was carried out in the context of Kinshasa, DR Congo in 2005–6. As a point of comparison, in 2009–2011, the prevalence of malaria parasitaemia in children six to 59 months old in Kinshasa was an average of 6.4 %, with higher levels reported in less urban areas [21]. The prevalence of malaria in the current study population varied from 21 % prevalence in early pregnancy to 9 % at delivery, which is low compared to previous studies of malaria in pregnancy in endemic areas [3, 22]. The relatively low proportion of positive samples at delivery can largely be explained by two-dose IPTp and treatment of microscopic malaria infection throughout the study.

Because it is ultimately important to be able to compare different diagnostics, LCA was used to compare the four tests in an agnostic fashion. LCA has previously been used to compare PCR and histopathology among smear-negative pregnant women in Malawi [23]. LCA does not assume a gold standard but constructs one based on the results of the individual tests [10]. LCA assumes that diagnostic tests are conditionally independent, given the true disease status of the subject. Under the assumption of the model, the joint likelihood for the diagnostic tests can be developed and parameters are estimated by maximum likelihood using an expectation-maximization type procedure [17]. While LCA assumes test independence, it is robust to violations of this assumption [24]. Based on the LCA, all tests were relatively specific (>95 %) but IHC and PCR had the highest sensitivities (68 and 56 %, respectively). Since IHC can detect parasites when they sequester in the placenta but do not circulate, while PCR detects extremely low parasitaemias, the combination of both methods might provide a means of detecting the greatest number of infections.

## Conclusions

In summary, these results suggest that PCR and/or IHC may be appropriate diagnostic methods for malaria in pregnant women in areas where high-quality processing of placental tissue is difficult.

## Abbreviations

PCR: polymerase chain reaction
qPCR: quantitative polymerase chain reaction
IHC: immunohistochemistry
HRP2: histidine-rich protein 2
DRC: Democratic Republic of Congo
LCA: latent class analysis
UNC: University of North Carolina at Chapel Hill
